# A tumor-like renal arteriovenous malformation on ^18^F-PSMA-1007 PET/CT: a case report

**DOI:** 10.3389/fmed.2024.1420473

**Published:** 2024-05-31

**Authors:** Yaqi Feng, Wenjiang Zhao, Yawen Feng, Wenli Dai

**Affiliations:** ^1^Department of Nuclear Medicine, the First College of Clinical Medical Science, China Three Gorges University, Yichang, Hubei, China; ^2^Department of Interventional Radiology, the First College of Clinical Medical Science, China Three Gorges University, Yichang, Hubei, China

**Keywords:** arteriovenous malformation, ^18^F-PSMA-1007, ^18^F-FDG, PET/CT, renal angiography

## Abstract

**Background:**

Renal arteriovenous malformations (rAVMs) are congenital abnormal pathways between renal arteries and veins that are rare in the general population. It is often misdiagnosed as malignant renal tumors with abundant blood supply, and the definitive diagnosis primarily relies on angiography. Multimodality imaging, including contrast-enhanced computed tomography (CT), magnetic resonance imaging (MRI), and positron emission tomography (PET)/CT plays an important role in the differential diagnosis of renal space-occupying lesions.

**Case presentation:**

A 56-year-old man presented with abdominal distension, loss of appetite, and back pain without obvious cause 2 years ago, without nausea vomiting, or frequent urination. Gastroscopy and colonoscopy showed multiple polyps in the duodenum and colon. Abdomen contrast-enhanced CT revealed a mass of 1.6 × 1.4 cm in the left kidney, which was considered to be a malignant tumor. PET/CT was performed for further diagnosis; the ^18^F-fluorodesoxyglucose (^18^F-FDG) PET/CT scan showed mild uptake in the left renal mass, while no uptake of ^18^F- prostate-specific membrane antigen (PSMA) was observed. Following a multidisciplinary discussion, the possibility of renal AVMs was considered and subsequently confirmed by renal angiography as the diagnosis. Then, selective segmental renal artery embolization was performed for treatment.

**Conclusion:**

Renal AVMs are extremely rare in clinical practice. Due to limited research on the application of ^18^F-FDG and ^18^F-PSMA PET/CT to renal AVMs, its role remains largely unexplored. With the increasing popularity of PET/CT imaging, comprehensive imaging of the disease has become indispensable. We report the first case of PSMA PET/CT imaging in renal AVMs, and when PSMA expression is absent in a renal mass, the possibility of renal AVMs should be considered.

## Introduction

Renal AVMs are congenital abnormal pathways between renal arteries and veins. They are rare diseases with a morbidity of less than 0.04% in the general population (1). The typical presentation of patients is the presence of hematuria or incidental detection during diagnostic imaging. It can be divided into congenital and acquired types (2). Congenital renal AVM can be divided into 3 categories: cirsoid, angiomatous, and aneurysmal (3). The acquired causes mainly include trauma, surgery, percutaneous renal biopsy, vasculitis, and renal malignant tumors (4). The main purpose of imaging is to establish a definitive diagnosis of the target lesion and differentiate renal AVM from other renal lesions. Imaging such as ultrasound, computed tomography angiography (CTA), magnetic resonance angiography (MRA), and renal arteriography can differentiate types of vascular malformations based on the predominant vessel type, anatomy, and flow patterns. However, current imaging modalities fail to assess the biological activity of the vascular malformation. ^18^F-FDG-PET/CT is a functional imaging modality that plays a considerable role in the diagnosis of malignancies while also serving as a surrogate marker for angiogenesis through increased FDG uptake, thereby aiding in the localization of lesions (5). PSMA is expressed in neovascularization, including tumor angiogenesis and nonmalignant situations (6); However, its application in renal AVMs has not been reported yet.

Therefore, we reported the diagnosis and treatment of a patient with renal AVM and demonstrated the unique features of ^18^F-PSMA-1007 and ^18^F-FDG uptake in renal AVMs.

## Case description

A 56-year-old male presented with unexplained abdominal distension 2 years ago, accompanied by easy satiety, a loss of appetite, and back pain. Physical examination showed no abnormalities. Laboratory tests for liver and kidney function as well as tumor markers were normal. Gastroscopy revealed the presence of duodenal polyps, while colonoscopy showed multiple polyps in the colon and rectum. After the polypectomy, pathology showed duodenal bulb and sigmoid colon polyps and a rectal tubular adenoma. The patient had a previous history of tonsillitis resection.

**Figure 1:**
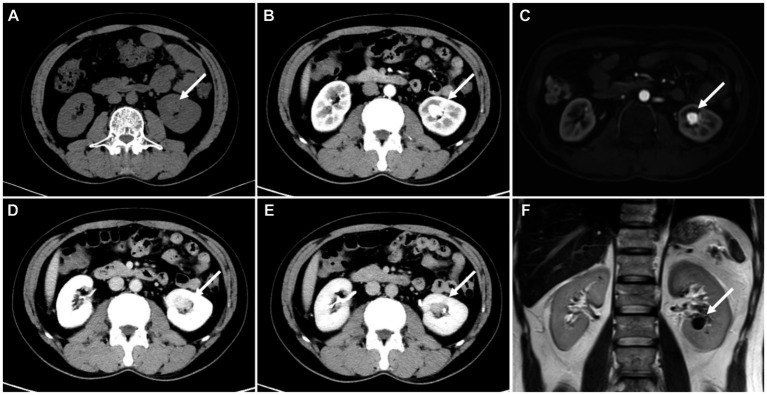
**(A)** CT showed a 1.6 cm × 1.4 cm nodule in the left kidney proximal to the renal hilum (white arrow). **(B)** The arterial phase of the nodule showed marked enhancement (mean 202HU), conspicuously decreased during the **(D)** venous phase (mean 160HU) and **(E)** delayed phase (mean 123HU). **(C)** Enhanced MRI showed significant enhancement of the nidus. **(F)** T2 displayed the flowing void effect of the nidus.

The patient underwent a CT examination of the abdomen, revealing a 2.5 × 1.9 cm low-density lesion with unclear boundaries in the right lobe of the liver. Contrast-enhanced CT was recommended. During contrast-enhanced CT scanning of the abdomen, the lesion in the right lobe of the liver showed no obvious enhancement, which suggested a complicated cyst. Additionally, a soft tissue density nodule measuring 1.6 × 1.4 cm with a CT value of 33HU was found in the renal sinus in the lower pole of the left kidney. This nodule showed significant enhancement during the arterial phase (CT value: about 202HU) and conspicuously decreased during the venous phase (CT value: about 160HU), raising the possibility of clear cell renal cell carcinoma (ccRCC; Figure 1). MRI scan showed a 1.6 cm diameter nodule in the left renal sinus, which was isointensity on T1 weighted image and hypointensity on T2 weighted image and significantly enhanced in the arterial phase.

**Figure 2:**
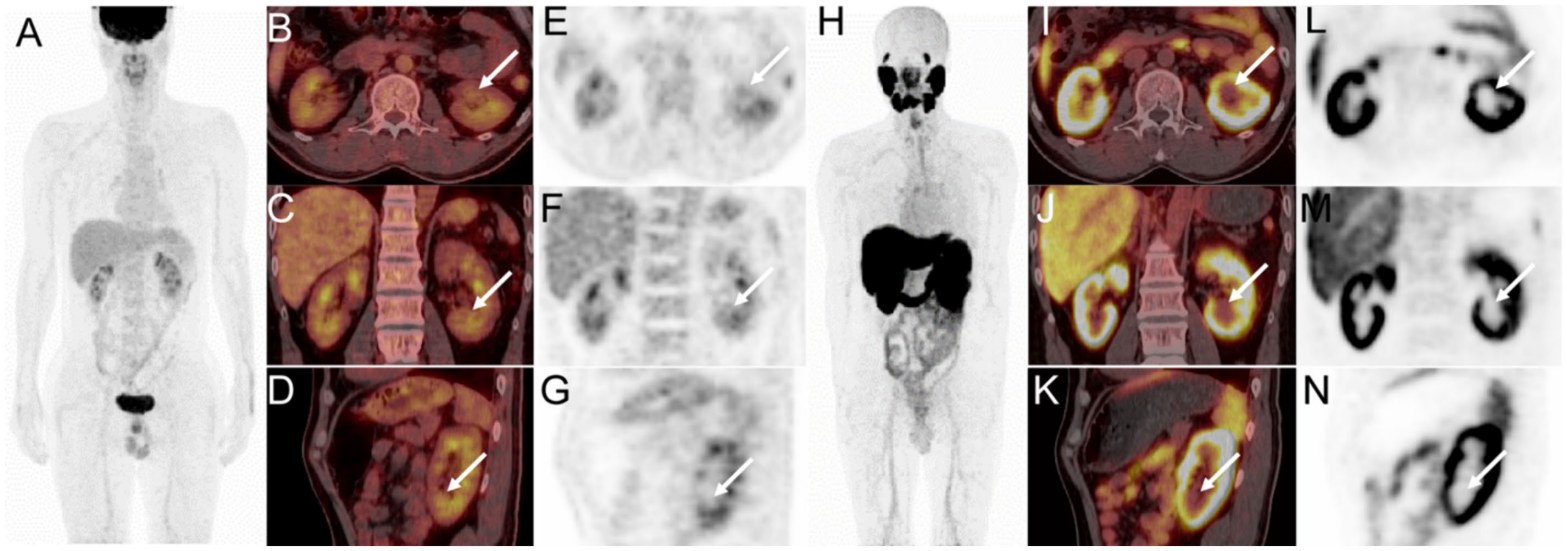
^18^F-FDG PET/CT images (**A**, MIP; **B**, axial fusion PET/CT; **C**, coronal fusion PET/CT; **D**, sagittal fusion PET/CT; **E**, axial PET; **F**, coronal PET; **G**, sagittal PET) showed mild FDG uptake in the renal mass (white arrow, SUVmax 4.5). ^18^F-PSMA-1007 PET/CT images (**H**, MIP; **I–K**, axial, coronal, and sagittal fusion PET/CT; **L–N**, axial, coronal, and sagittal PET) showed no tracer concentration in the left renal lesion (white arrow).

Subsequently, an ^18^F-FDG PET/CT was done to help determine the exact nature of the lesion in the left kidney. The lesion demonstrated mild FDG uptake with a maximum standard uptake value (SUVmax) of 4.5 (Figure 2). According to the low to moderate uptake of ^18^F-FDG, the following day, ^18^F-PSMA-1007 was carried out to evaluate tumor neovascularization. ^18^F-PSMA-1007 PET/CT scan showed no uptake in the left renal lesion (Figure 2). The absence of ^18^F-PSMA-1007 uptake in a renal mass made ccRCC less likely.

**Figure 3:**
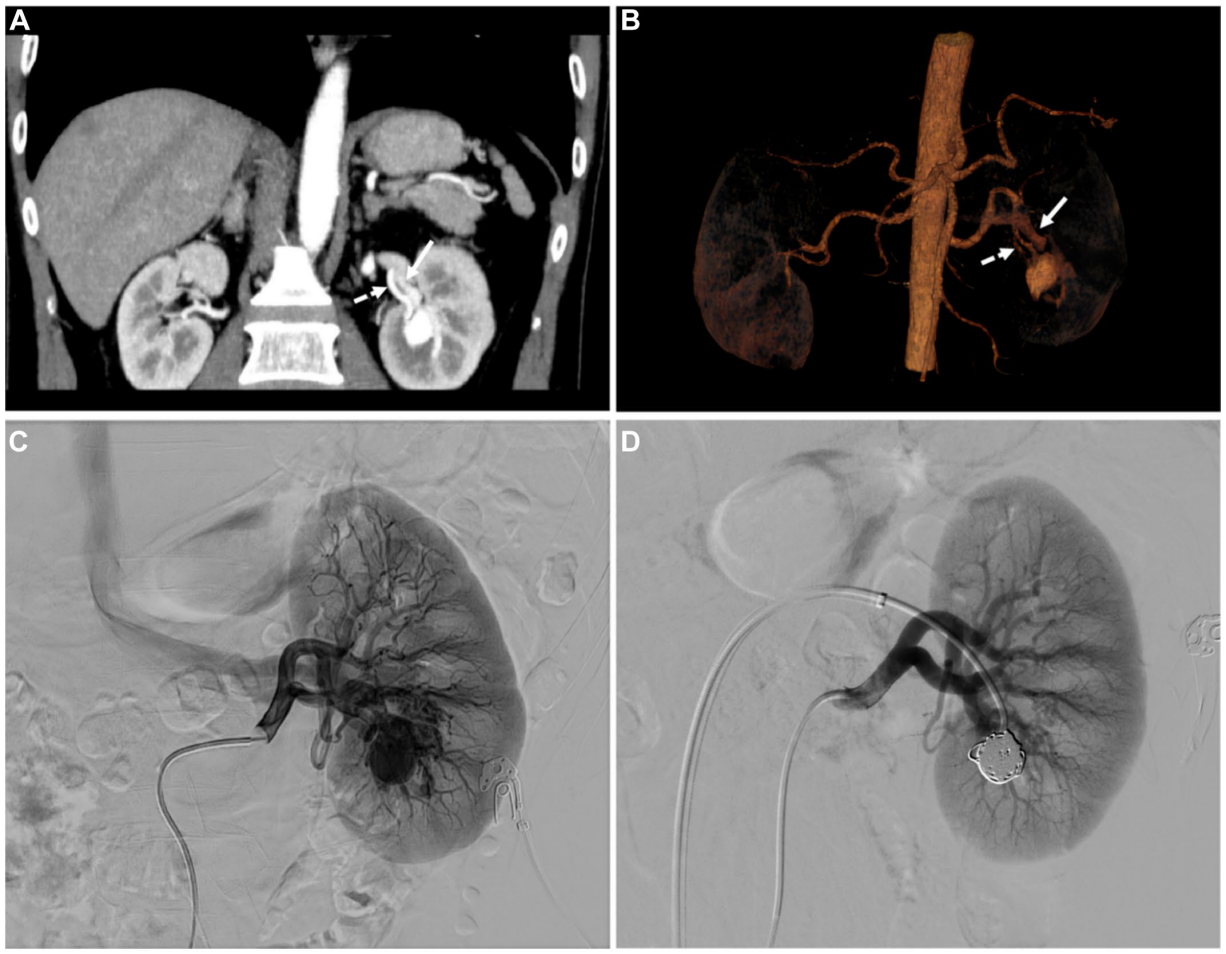
**(A)** The coronal MIP and **(B)** volume rending (VR) image displayed the left renal feeding artery (dashed arrows) and thickened draining vein (white arrows). **(C)** Renal angiography shows the renal aneurysmal sac located in the lower pole of the left renal, which is supplied by a branch of the left renal artery. Early drainage of contrast from the left renal vein is also remarkable. **(D)** After coil post-embolization, the aneurysmal sac vanished without the renal vein filling up.

After that, a multidisciplinary discussion between the department of radiology, nuclear medicine, and urology was carried out. Through analysis, it was found that the left renal lesion was significantly enhanced in the arterial phase and decreased significantly in the venous phase, and the degree of enhancement was always consistent with the abdominal aorta, considering the possibility of vascular malformation. By contrast enhanced CT reconstruction, maximum intensity projection (MIP), and volume rendering (VR) showed feeding arteries and thickened draining veins (Figure 3). Combined with PET/CT, there was no PSMA uptake and near mild FDG uptake, ccRCC can be excluded. Therefore, digital subtraction angiography (DSA) was recommended for further examination. Left renal angiography demonstrated a renal aneurysmal sac, and early drainage of contrast from the left renal vein was also remarkable. After coil post-embolization, the aneurysmal sac vanished without the renal vein filling up (Figure 3). The patient was cured and discharged after treatment.

## Discussion

Renal AVMs can be classified into congenital or acquired. In the congenital category, renal AVMs can be divided into three morphological classes: cirsoid, angiomatous, and aneurysmal. Our case is the cirsoid type. The cirsoid type is more common, the anomalous blood vessels of the cirsoid type are typically situated within the submucosal lamina propria of the collecting system, exhibiting aberrant connections between segmental or interlobar arteries and veins. The abnormally dilated blood vessels manifest as clumps, plexiform, or beaded formations. The angiomatous type is distinguished by a single big artery feeding many linked distal branches and draining veins. Aneurysmal type has one or more big aberrant blood vessels that are directly connected between the intrarenal artery and vein (7). Renal AVMs are rare causes of hematuria, and other symptoms include backache, renovascular hypertension, and high-output cardiac failure. Most patients do not have symptoms until the age of 35, with the majority of cases occurring in the elderly, which may be related to the fact that the walls of the blood vessels become weaker (8).

There are a variety of imaging methods that can be used to diagnose and evaluate AVMs. Ultrasound can be used as the first choice for the AVMs, and preliminary screening can be done, but the resolution is low, and not easy to show blood tubes, resulting in low accuracy. Contrast-enhanced CT can provide rich information for the diagnosis of AVMs, which can better display the location of the lesion, blood flow status, and adjacent tissue involvement. In addition, CT image post-processing techniques such as MIP, multi-planar reformation (MPR), and VR techniques allowed for a more intuitive visualization of vascular malformations and drainage vessels from multiple perspectives. AVMs lesions can show abnormal mixed signals on MRI and there is an obvious flow void effect. Renal arteriography is considered the gold standard for diagnosing renal AVMs (2). This examination provides direct visualization of the precise location, blood supply, and hemodynamic alterations associated with AVMs, including even minute lesions. Additionally, it enables simultaneous treatment alongside accurate diagnosis.

Initially, the renal mass was suspected to be ccRCC due to its abundant blood supply. PET/CT can be used for the differential diagnosis of benign and malignant renal tumors. To confirm the diagnosis, PET/CT is recommended. FDG can reflect the level of glucose metabolism and can be used for the differential diagnosis of malignant tumors. Among the blood-rich renal masses, ccRCC and lipid-poor angiomyolipoma are the most common and require differential diagnosis. Zhao et al. showed that the median SUVmax of ccRCC was 4.1 (2.7–9.5), and the SUVmax of high-grade ccRCC was significantly different from that of low-grade (median SUVmax 3.1 vs. 7.3, *p* < 0.001) (9). Lin et al. investigated 21 patients with renal angiomyolipoma who underwent FDG PET/CT and discovered that renal angiomyolipoma had low uptake on FDG PET/CT (SUVmax 0.81–1.98) (10). Sometimes there is an overlap in FDG uptake between ccRCC and renal angiomyolipoma, which is somewhat difficult to distinguish. Unlike most malignant tumors, the use of FDG in RCC is of limited value for several reasons: (1) FDG is excreted by the kidney, which leads to a low tumor background ratio; (2) downregulation of glucose transporter 1 (GLUT1) expression leads to low FDG-avidity; (3) Different RCC subtypes have different FDG-avidity. Nevertheless, FDG has a high accuracy in the diagnosis of RCC metastasis and can be used to detect RCC metastases and to assess treatment response (11). For vascular malformations, Angiogenesis is present in both tumor growth and the creation of nidus. Increased FDG uptake in tumor angiogenesis has been verified by studies (12). A case report demonstrated that high-flow AVMs can exhibit a high FDG uptake (5). In our case, the FDG metabolism of the nidus was not high, which may be related to the low blood flow of the AVMs in our case.

PSMA, a type II transmembrane glycoprotein, is overexpressed in prostate cancer epithelial cells and is also expressed in neovascular endothelial cells in other solid tumors, including RCC (13, 14). In normal renal parenchyma, PSMA is mainly expressed in proximal tubular epithelial cells. a meta-analysis showed that the pooled sensitivity and specificity of PSMA PET/CT for detection of RCC local lesions were 87.2%, 100% and metastatic disease were 92%, 96.9% (15). Some reports have demonstrated the usefulness of ^18^F-PSMA-1007 PET/CT in the diagnosis and initial staging of RCC. In these cases, the primary renal tumor had a significant tracer concentration (16, 17). In our case, the absence of PSMA expression in a renal mass would reduce the likelihood of ccRCC.

PSMA can be expressed on the endothelial cell membrane of neovascularization, so some benign lesions can also show PSMA uptake (18, 19). Some hemangiomas such as splenic hemangioma (20, 21), hepatic hemangioma (22), subcutaneous lobular capillary hemangioma (23), and vertebral hemangioma (24), have been shown to have a significant PSMA uptake in some published cases. However, PSMA uptake in renal AVMs has not been reported. We report for the first time the imaging of PSMA PET/CT in renal AVMs, and our imaging shows that PSMA may not be expressed in renal AVMs. The possible reason for the lack of PSMA uptake in renal AVMs in our case is that the renal AVM is congenital in formation, and there is no neovascularization, so it does not express PSMA. The underlying cause for the absence of PSMA uptake in renal AVMs remains uncertain and requires further investigation.

It is important to distinguish renal AVMs from renal carcinoma, Lipid-poor angiomyolipoma, renal artery aneurysms, and so on. ccRCC occurs in the renal parenchyma, the enhancement is obvious in the arterial phase, but the degree of enhancement is less than that of blood vessels. The lesions decrease significantly in the venous phase, and secretory phase and there are no malformation of vascular mass and thickening of feeding arteries and draining veins. In our case, the lesion was located in the renal sinus and was markedly enhanced in the arterial phase; the degree of enhancement was consistent with that of the abdominal aorta. Moreover, there was no significant uptake of FDG and PSMA, so it can be distinguished from renal carcinoma. Lipid-poor angiomyolipoma can exhibit a “fast in and fast out” or gradual enhancing pattern, depending on the level of vascular growth. The enhancing pattern of lipid-poor angiomyolipoma overlaps with ccRCC, and their FDG metabolism also overlaps, making it difficult to identify. Renal artery aneurysms most commonly occur at the bifurcation of the renal artery, and the aneurysm is enhanced, without malformed vascular masses and draining veins (25).

The key to the treatment of renal AVMs is to relieve venous hypertension. The treatment methods include interventional embolization, surgery, and so on. Surgical treatment is invasive and is recommended only for patients with anatomical abnormalities or hemodynamic instability, with the risk of loss of renal function (8). Interventional therapy has the advantages of minimal trauma, good curative effect, and maximum preservation of normal renal tissue (26). The main purpose of interventional treatment of renal AVMs is to eliminate the “nest” and re-drain blood flow to normal vessels. In our case, the patient ultimately accepted coil embolization therapy. After the operation, the aneurysmal sac completely vanished without any filling of the renal vein. One year after discharge, the patient provided feedback indicating that he had no discomfort such as lumbago and back pain, and CTA suggested occlusion of the renal AVM.

## Conclusion

We report a case of a patient with renal AVMs which are rare disease and their imaging features are similar to that of renal tumors, so an accurate diagnosis is essential. PET/CT can be used for the differential diagnosis of renal masses. In this case, the absence of PSMA expression in a renal mass would reduce the likelihood of RCC. We report the first case of PSMA PET/CT in renal AVMs, the mechanism by which renal AVMs do not express PSMA requires further investigation. Our case highlights the possible uses of PSMA PET/CT for the assessment of renal masses and the possibility of renal AVMs being considered when PSMA uptake is absent. Renal angiography is the gold standard for the diagnosis of renal AVMs, and interventional therapy is the first choice for the treatment. Further prospective study is recommended.

## Data availability statement

The raw data supporting the conclusions of this article will be made available by the authors, without undue reservation.

## Ethics statement

The studies involving humans were approved by Ethics Committee of Yichang Central People’s Hospital. The studies were conducted in accordance with the local legislation and institutional requirements. The participants provided their written informed consent to participate in this study. Written informed consent was obtained from the patient for the publication of any potentially identifiable images or data included in this article.

## Author contributions

YqF: Writing – original draft, Writing – review & editing. WZ: Methodology, Writing – review & editing. YwF: Data curation, Writing – review & editing. WD: Writing – review & editing.
